# Transcriptional profiling analysis and functional prediction of long noncoding RNAs in cancer

**DOI:** 10.18632/oncotarget.6993

**Published:** 2016-01-23

**Authors:** Jiao Yuan, Haiyan Yue, Meiying Zhang, Jianjun Luo, Lihui Liu, Wei Wu, Tengfei Xiao, Xiaowei Chen, Xiaomin Chen, Dongdong Zhang, Rui Xing, Xin Tong, Nan Wu, Jian Zhao, Youyong Lu, Mingzhou Guo, Runsheng Chen

**Affiliations:** ^1^ Key Laboratory of RNA Biology, Institute of Biophysics, Chinese Academy of Sciences, Beijing 100101, China; ^2^ Beijing Key Laboratory of Noncoding RNA, Institute of Biophysics, Chinese Academy of Sciences, Beijing 100101, China; ^3^ University of Chinese Academy of Sciences, Beijing 100049, China; ^4^ Department of Gastroenterology and Hepatology, Chinese PLA General Hospital, Beijing 100853, China; ^5^ Laboratory of Molecular Oncology, Key Laboratory of Carcinogenesis and Translational Research (Ministry of Education), Peking University Cancer Hospital and Institute, Beijing 100142, China; ^6^ PLA General Hospital Cancer Center Key Laboratory, Medical School of Chinese PLA, Beijing 100853, China; ^7^ International Joint Cancer Institute, the Second Military Medical University, Shanghai 200433, China

**Keywords:** IncRNA, expression, biomarker, gastric cancer, colon cancer

## Abstract

Long noncoding RNAs (lncRNAs), which are noncoding RNAs (ncRNAs) with length more than 200 nucleotides (nt), have been demonstrated to be involved in various types of cancer. Consequently, it has been frequently discussed that lncRNAs with aberrant expression in cancer serve as potential diagnostic biomarkers and therapeutic targets. However, one major challenge of developing cancer biomarkers is tumor heterogeneity which means that tumor cells show different cellular morphology, metastatic potential as well as gene expression. In this study, a custom designed microarray platform covering both mRNAs and lncRNAs was applied to tumor tissues of gastric, colon, liver and lung. 316 and 157 differentially expressed (DE-) protein coding genes and lncRNAs common to these four types of cancer were identified respectively. Besides, the functional roles of common DE-lncRNAs were inferred based on their expression and genomic position correlation with mRNAs. Moreover, mRNAs and lncRNAs with tissue specificity were also identified, suggesting their particular roles with regard to specific biogenesis and functions of different organs. Based on the large-scale survey of mRNAs and lncRNAs in four types of cancer, this study may offer new biomarkers common or specific for various types of cancer.

## INTRODUCTION

Cancer has been a major health problem worldwide, with an estimate of more than 4,500 new cases each day in 2014 [1]. Numerous factors, including environment [2], lifestyle [3] and genetic defects [4, 5], contribute to tumorigenesis and development of cancer. The development of high-throughput profiling technology has enabled characterization of cancer cells from perspective of genome, epigenome and transcriptome [6, 7]. Several studies have succeeded in identifying tumor biomarkers for cancer detection, diagnosis or prognosis determination for specific types of cancer, such as estrogen receptor and progesterone receptor in breast cancer [8] and prostate-specific antigen in prostate cancer [9]. Although high heterogeneity was observed between the transcriptomic landscape of distinct types of cancer [10], cancer cells share characteristics such as dys-regulated cell growth and potential to invade compared to normal cells [11]. Consequently, biomarkers might be either specific to a particular type of cancer or general to multiple types of cancer.

The majority of previous efforts have focused on protein coding genes (PCGs). However, since lncRNAs have been implicated to play important roles in multiple biological processes such as cell cycle [12], cell apoptosis [13] as well as signal pathway [14, 15], lncRNAs might function as tumor suppressors or oncogenic drivers [16]. Dys-regulated expression of some lncRNAs, such as *HOTAIR*, *PCAT1* and *SNHG1*, has been considered as indicator of several human cancers [17–19]. However, the molecular mechanism of lncRNA functions in cancer biology is still poorly understood.

In this study, microarray test was applied to obtain expression profiles of both protein coding genes and lncRNAs in tumor and paired adjacent non-tumor tissues from 76 patients (20 with gastric cancer, 20 with colon cancer, 16 with liver cancer and 20 with lung cancer). The microarray platform is custom designed covering both kind of transcripts of 21,789 mRNAs and 39,311 lncRNAs. A collection of 157 lncRNAs as well as 316 PCGs with dys-regulated expression in tumor tissues compared with adjacent non-tumor tissues in all of four types of cancer was observed. The possible functions of the identified 157 common DE-lncRNAs were further inferred based on their correlation with PCGs from both perspective of expression and genomic coordinates. Besides, PCGs and lncRNAs whose expression showed tissue specifity in any type of cancer were also identified. Part of the results were validated by quantitative PCR (qPCR) in external patient samples. In summary, this study has discovered cancer- and tissue- associated PCGs and lncRNAs through integrative analysis of expression profile revealed by custom designed microarray, thus providing a systematic summary of expression pattern and biological relevance of lncRNAs in cancer.

## RESULTS

### Identification of PCGs and lncRNAs as candidate common biomarkers for cancer

**Figure 1:**
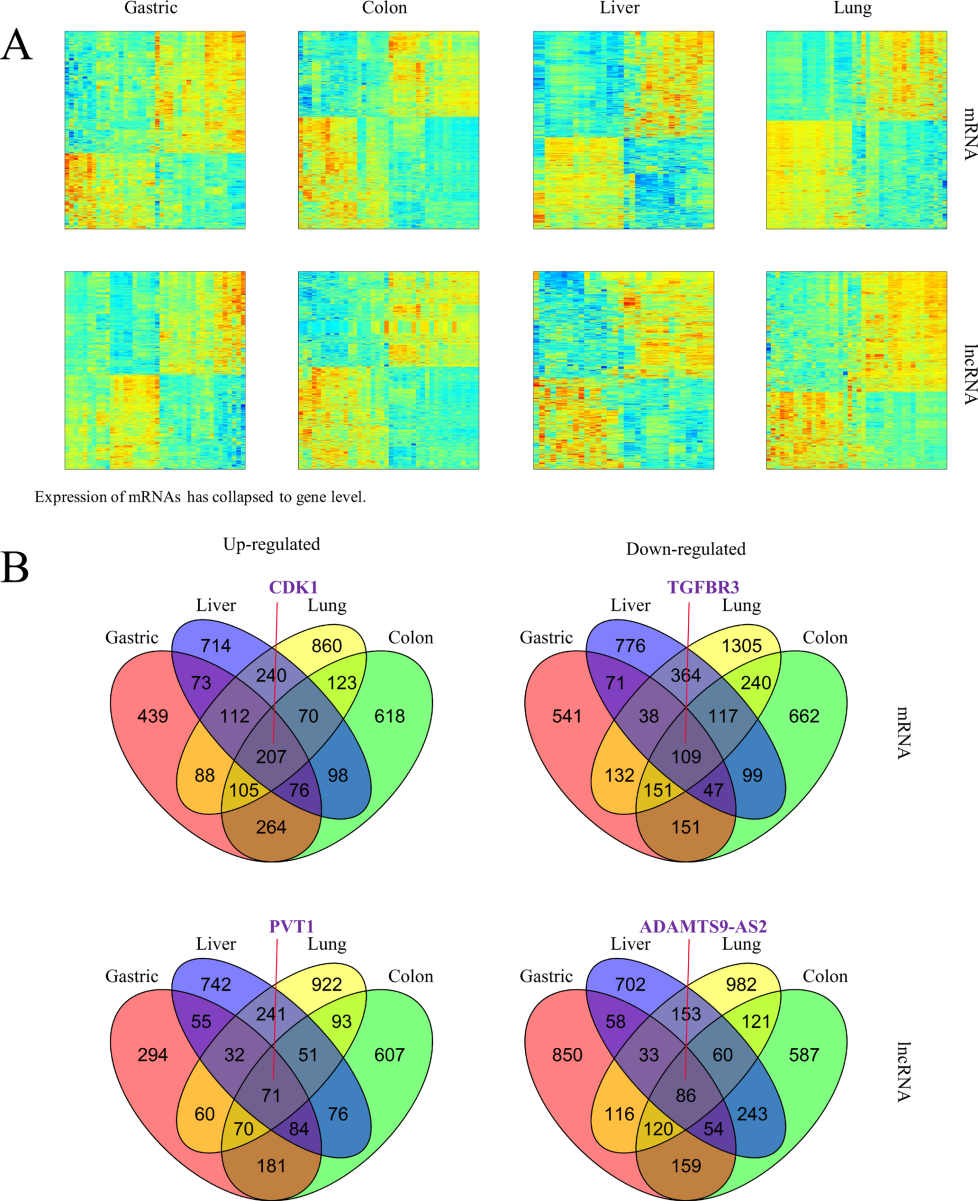
Altered expression of mRNAs and lncRNAs across cancer types (**A**) Hierarchically clustered heatmaps of mRNAs and lncRNAs that are differentially expressed (student *t*-test corrected *p*-value < 0.05 and fold change > 1.5) in each type of cancer tissues compared to adjacent non-cancerous tissues. (**B**) Venn diagrams showing up-regulated and down-regulated mRNAs and lncRNAs whose dys-regulated expression pattern was shared by four types of cancer. Literature curated cancer biomarkers were indicated as examples.

The global expression profile of both PCGs and lncRNAs in four types of cancer tissues (gastric, colon, liver, and lung) and adjacent non-cancerous tissues were examined by a custom microarray platform (see Materials and Methods for details). Hundreds of PCGs and lncRNAs show differential expression in each type of cancer (Figure 1A).

**Figure 2:**
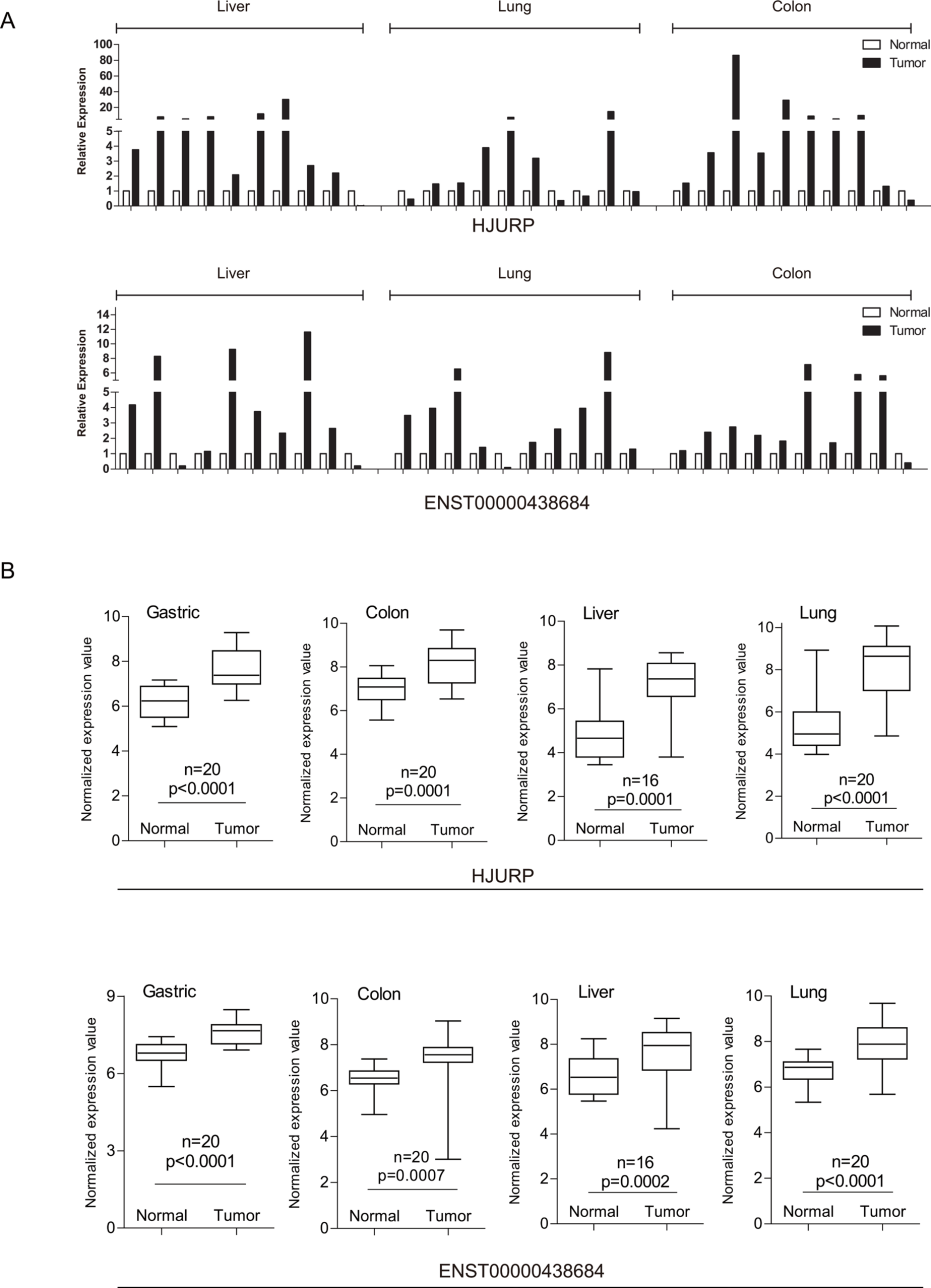
qRT-PCR validation of lncRNA and mRNA expression in samples from external patients (**A**) Expression of common DE-mRNAs/lncRNAs (upper panel representing a mRNA and lower panel representing a lncRNA). (**B**) Boxplots represent common DE-mRNAs/lncRNAs' expression based on the microarray data. (upper panel representing a mRNA and lower panel representing a lncRNA).

We first examined the expression patterns of several well-known cancer-related lncRNAs revealed by our microarray experiments [20]. Notably, 8 of the 12 lncRNAs examined showed differential expression between tumors and non-tumor tissues in at least one type of cancer (Supplementary Figure S1). However, they could hardly serve as biomarkers common to multiple types of cancer other than a few examples. Besides, several of them might produce confusion. For example, as shown in our data, *HOTTIP* was indicated to have potential oncogenic function in liver cancer and gastric cancer as it showed up-regulated expression in tumors compared to adjacent non-tumor tissues while its association with colon cancer and lung cancer was not found. *UCA1* is a lncRNA reported to promote cell proliferation in both breast cancer [21] and bladder cancer [22]. Of the cancer types used in our microarray experiments, *UCA1* showed its oncogenic potential in gastric, colon and lung but had expression characteristic of tumor suppressor in liver. Although such lncRNAs might explain complex and heterogeneous nature of different cancer types [23], it would be more desirable to discover PCGs or lncRNAs as indicators in various types of cancer. As a consequence, further efforts were taken to identify PCGs and lncRNAs with up-regulated or down-regulated expression in all of four cancer types (Student *t*-test, false discovery rate (FDR) < 0.05, fold change (FC) > 1.5), resulting in 207 PCGs and 71 lncRNAs as potential oncogenes and 109 PCGs and 86 lncRNAs as potential tumor suppressors common to these four cancer types (Figure 1B, Supplementary File 1). These include some known cancer biomarkers such as PCGs *CDK1* [24, 25] and *TGFBR3* [26], as well lncRNAs *PVT1* [27, 28] and *ADAMTS9-AS2* [29]. In order to validate the alterations of PCG and lncRNA expression obtained from microarray data, we validated a subset of them across a panel of external samples by qRT-PCR (Figure 2A; the validation in gastric cancer was absent due to lack of additional samples). The qRT-PCR result showed high consistency with microarray data (Figure 2B).

**Figure 3:**
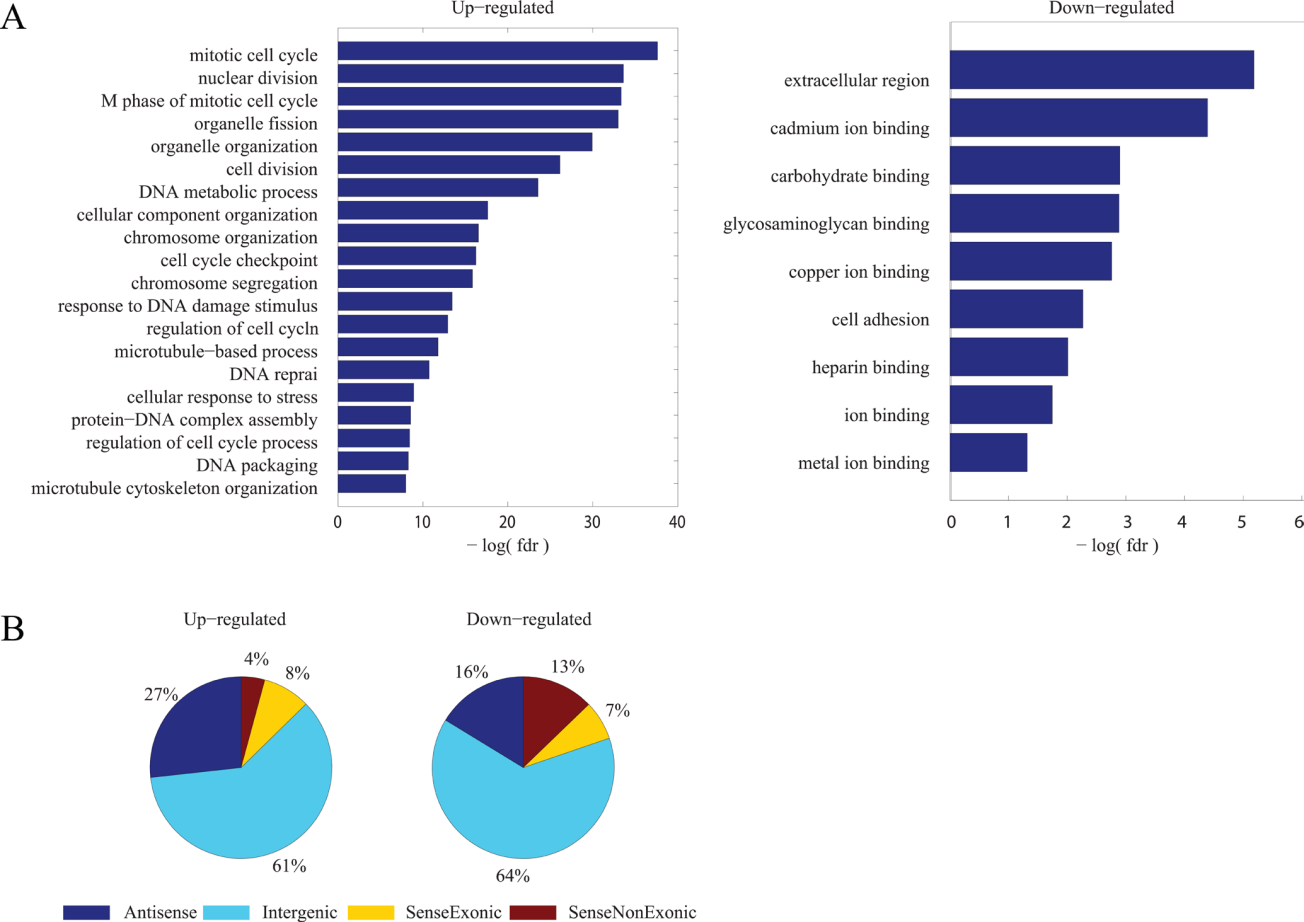
Signatures of common dys-regulated mRNAs and lncRNAs (**A**) Functional enrichment result by DAVID for common up-regulated and down-regulated PCGs respectively. The resulted GO terms of biological processes with a FDR < 0.1 were considered statistically significant and depicted. (**B**) Distribution of common up-regulated and down-regulated lncRNAs according to their genomic context association with PCGs.

Next, gene ontology enrichment analysis was performed in the common DE-PCGs (Figure 3A). Up-regulated PCGs were enriched in cell cycle related biological processes while down-regulated PCGs were associated with cell adhesion, consistent with the common characteristics of cancer which refer to promoted cell proliferation and activated cell migration. Similarly, it could be inferred that common DE-lncRNAs might also play important roles in regulating essential biological processes and dys-regulated expression of them would lead to abnormality.

Classifying lncRNAs into different subgroups according to their genomic context association with PCGs results in the largest subgroup to be intergenic lncRNAs [30, 31]. Similar proportion of different subgroups was observed in these 157 common DE-lncRNAs (Figure 3B).

### Functional prediction of common DE-lncRNAs

**Figure 4:**
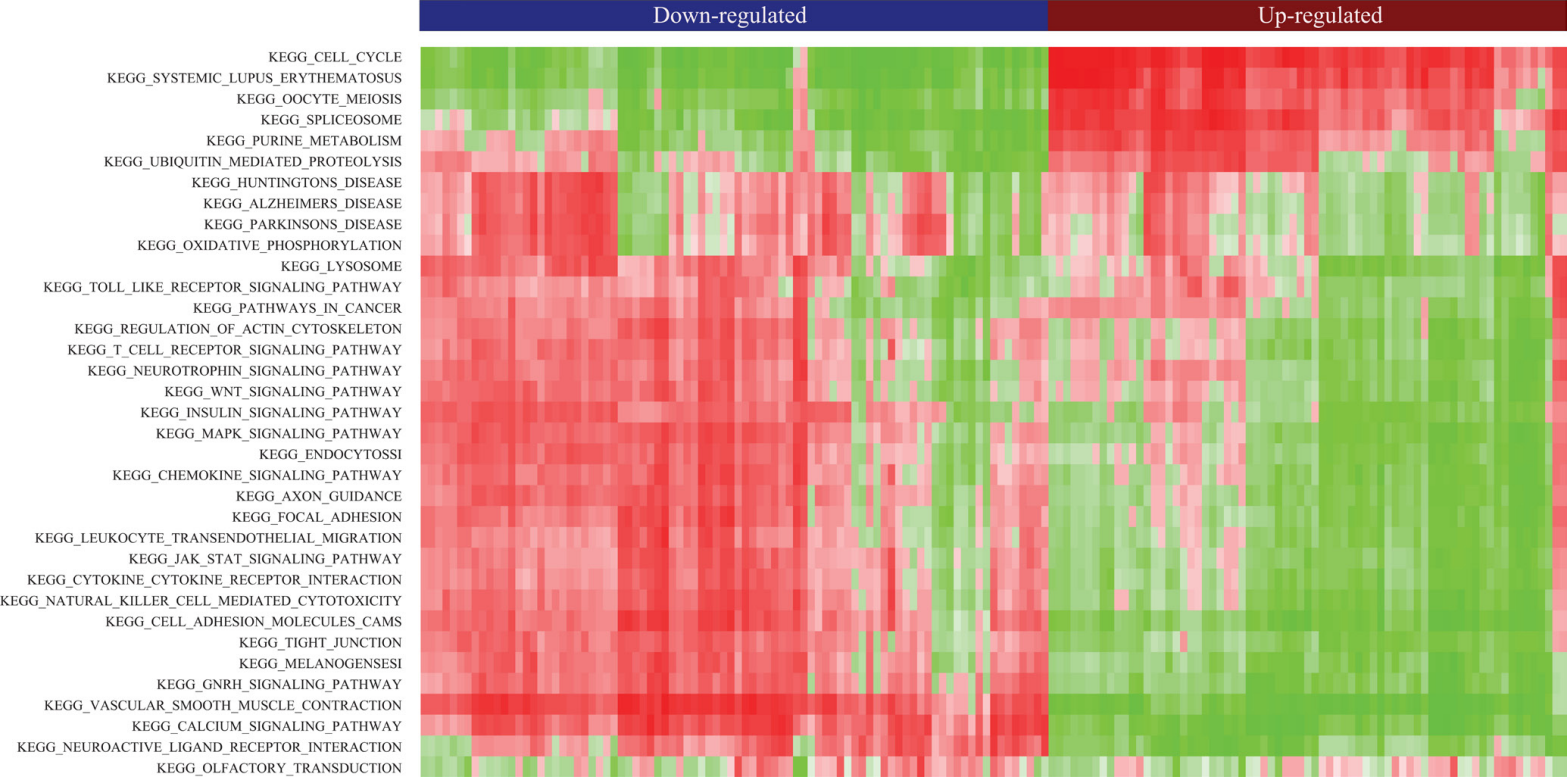
Heatmap of clustered pathway enrichment scores for common DE-lncRNAs Red (green) denotes positive (negative) nominal enrichment scores in gene set enrichment analysis (GSEA) for KEGG pathways.

Gene Set Enrichment Analysis (GSEA) [32, 33] was performed in order to gain insights into the biological significance of the identified DE-lncRNAs which might serve as biomarkers common to four types of cancer. Pearson correlation coefficients between expression profiles of mRNAs and common DE-lncRNAs across all tissues were calculated based on which common DE-lncRNAs associated gene sets were identified. Unsupervised hierarchical clustering of enrichment score of KEGG pathways clearly separated common up-regulated lncRNAs from common down-regulated lncRNAs (Figure 4). Particularly, the majority of common up-regulated lncRNAs were significantly associated with cell cycle, similar to the function enrichment result of common up-regulated PCGs. Besides, some of the common up-regulated lncRNAs were functionally related to spliceosome. The signaling pathways including WNT pathways and MAPK pathways, which these common DE-lncRNAs might be involved in, were also indicated. Since our knowledge of lncRNAs had been far less than PCGs, the expression profile association between lncRNAs and PCGs would be beneficial clues to understand functions of lncRNAs.

**Figure 5:**
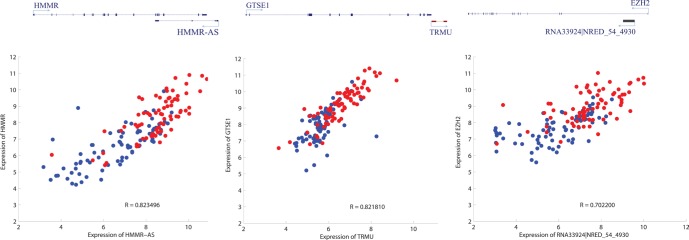
Three examples depicting potential cis regulation of common DE-lncRNAs on mRNAs Upper panel is simplified schematic diagram representing the relative genomic location of lncRNAs and their neighboring PCGs (left: antisense; middle: intergenic; right: intronic); lower panel is scatter plot characterizing the expression profile correlation of the pair of genomic interacting lncRNA and mRNA (red and blue representing cancer tissues and adjacent non-cancerous tissues respectively).

Another aspect of association between lncRNAs and PCGs is about genomic coordinates. Several lncRNAs have been known to function by cis-acting mechanism [34–36]. For example, *Kcnq1ot1* has a negative control of its neighboring PCGs [37, 38]. In order to figure out the possibility that the common DE-lncRNAs function in cis, the expression correlation between the common DE-lncRNAs and their genomic neighbor genes (the nearest PCGs for intergenic lncRNAs, the PCGs on the opposite strand of antisense lncRNAs, the host PCGs of intronic lncRNAs and overlapping PCG for sense-exonic lncRNAs) was investigated. Compared with the expression correlation with neighbor PCGs of genome-wide lncRNAs covered by the microarray platform, there is a significant positive correlation for that of common DE-lncRNAs (Supplementary Figure S2). Examples representing common DE-lncRNAs which might have a role of regulating gene expression in cis were also shown (Figure 5).

**Figure 6:**
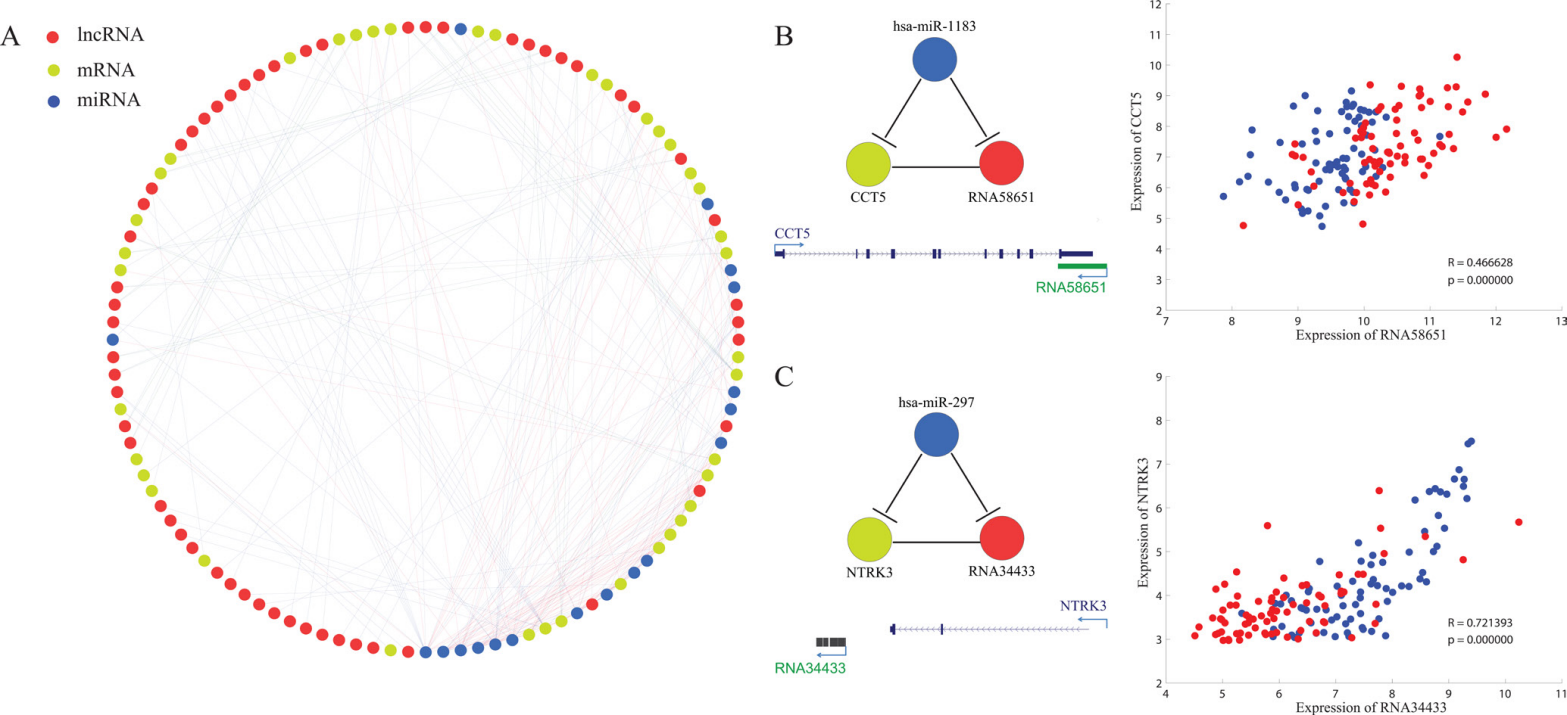
Prediction of lncRNAs as ceRNAs of their cis mRNAs (**A**) Tri-color network consisting of lncRNAs (red), mRNAs (yellow) and miRNAs (blue). (**B** and **C**) Two examples showing the minimal ceRNA motif (In the scatter plot, red and blue representing cancer tissues and adjacent non-cancerous tissues respectively, same with that of Figure 5) (B) an antisense lncRNA with oncogenic expression profile. (C) an intergenic lncRNA with tumor suppressor-like expression profile.

Some lncRNAs are reported to play important roles in tumorigenesis by acting as competing endogenous RNAs (ceRNAs) [39–42]. In order to explore the possibility that lncRNAs might regulate the expression level of neighboring PCGs via ceRNA pathway, a tri-color network was constructed to elucidate interactions among common DE-lncRNAs, neighboring PCGs and miRNAs (Figure 6A). Interactions between common DE-lncRNAs and neighboring PCGs involved both their genomic position interaction and expression profile correlation (correlation coefficient > 0.45 and *p*-value < 0.01) while miRNA targets prediction result by miRanda [43, 44] linked miRNAs and DE-lncRNAs or neighboring PCGs. Minimum tri-color submotifs were identified. For example, *CCT5* and its antisense lncRNA (*RNA58651* nominated in-house) had positively correlated expression profile and both of them were predicted to be targeted by *miR-1183* (Figure 6B). Another example is *RNA34433*, a novel intergenic lncRNA located downstream of *NTRK3*. *RNA34433* showed down-regulated expression in tumor tissues compared to adjacent non-tumor tissues in four types of cancer while *NTRK3* had been reported to be a potential tumor suppressor [45]. Both *RNA34433* and *NTRK3* were predicted to be targeted by *has-miR-297* (Figure 6C), whose potential role in cancer genesis should be further investigated. *MiR-34a*, a literature-curated tumor suppressor [46–48], had a higher degree than other miRNAs in the network. Moreover, a significant enrichment of experimentally determined miR-34a targets was obtained in neighboring PCGs of common up-regulated lncRNAs (Supplementary Figure S3) through Gene ontology enrichment analysis [49].

### Identification of tissue-specific PCGs and lncRNAs

**Figure 7:**
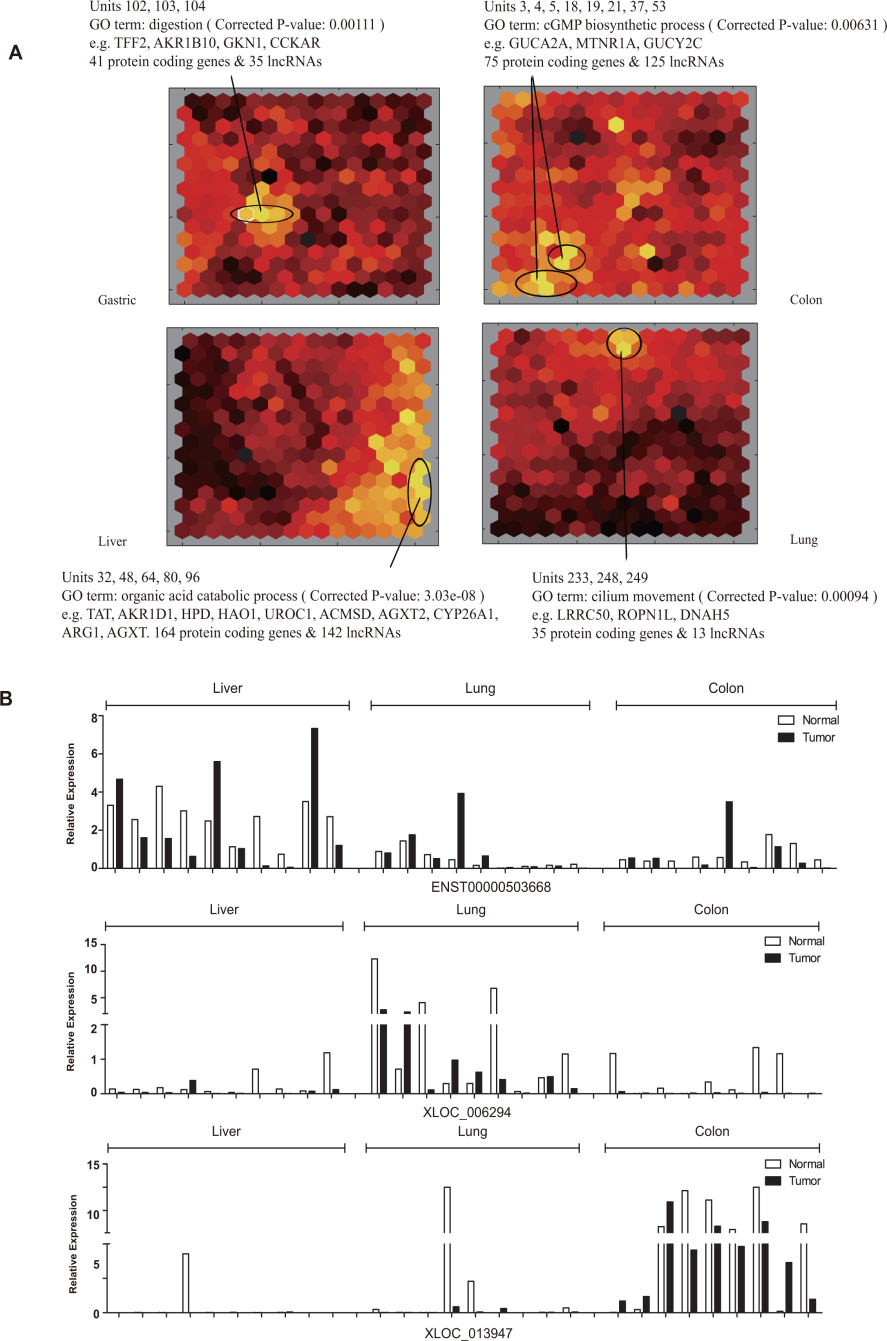
Transcriptome comparison by the self-organizing map (SOM) (**A**) Schematic illustration of expression profiles of four types of tissues depicted by SOM. Each hexagonal grid is a cluster of lncRNAs and PCGs (see also Figure S5). Grids showing significant expression specificity in each type of tissues were circled. Significantly enriched Gene Ontology (GO) terms for PCGs of circled clusters were indicated. (**B**) Expression of tissue-specific lncRNAs (upper, middle and lower panel representing an example of lncRNA specifically expressed in liver cancer, lung cancer and colon cancer, respectively).

Unsupervised hierarchical clustering of all tissues using common DE-lncRNAs apparently separated cancer tissues from adjacent non-cancerous tissues, but hardly distinguish different tissues (Supplementary Figure S4). Yet, higher degree of similarity between gastric and colon, as well as that between liver and lung, was observed in both cancer tissues and adjacent non-cancerous tissues, suggesting the indicative potential of lncRNAs for the origins and functions of different tissues. Consequently, a self-organizing map (SOM) based approach was applied in order to identify lncRNAs and mRNAs which might explain the specific characteristics of each type of gastric, colon, liver and lung tissues. lncRNAs and PCGs were first classified into hexagonal units, each of which represented a set of lncRNAs and PCGs whose expression profile are most similar to each other (Supplementary Figure S5). Then the color of each unit was assigned according to the overall expression level of lncRNAs and PCGs, brighter color representing higher expression while darker representing lower. Units with higher overall expression level in one type of tissues than others were determined to comprise lncRNAs and PCGs with tissue specificity which was subjected to later functional enrichment analysis (Figure 7A). Take gastric as an example, three units numbered 102, 103 and 104 respectively had obviously brighter color representing higher overall expression level in gastric tissues than other three types of tissues. An enrichment of digestion function was observed for the 41 PCGs, suggesting that the 35 lncRNAs in the same unit might also participate in the gastric-specific physiological process. Similarly, lncRNAs and PCGs with expression specificity in colon, liver and lung were identified respectively (see full list of tissue-specific lncRNAs and mRNAs in Supplementary File 2). qRT-PCR validation was performed on random selected tissue-specific lncRNAs and was in good agreement with microarray result (Figure 7B and Supplementary Figure S6).

## DISCUSSION

Previous efforts to study the pathogenesis of diseases have been focused on PCGs. However, the accumulating discoveries of lncRNA functions in various biological processes have revealed the potential of lncRNAs acting as cancer biomarkers. The majority of biomarker lncRNAs reported by now are derived from researches based on particular type of cancer, such as *SChLAP1* to be identified as a prostate cancer–associated lncRNA [50]. Considering that different types of cancer share common characteristics, we launched this study in order to identify lncRNAs with the potential to serve as common biomarkers for multiple types of cancer. By incorporating gastric, colon, liver and lung cancer tissues accompanied with paired control non-cancerous tissues into gene expression detection by a custom designed microarray platform covering 39,311 lncRNA transcripts, a total of 157 lncRNAs were identified as potential common biomarkers with expression pattern of either oncogenes or tumor suppressors. On the other hand, we also generated a list of lncRNAs and PCGs whose expression might explain the specific origins or functions of different tissues.

Despite the potential role of the identified common DE-lncRNAs as biomarkers general to different types of cancer, how the dys-regulation of their expression would prompt tumorigenesis remains a challenging problem. In this study, the association between lncRNAs and PCGs was established from the perspective of both expression correlation and genomic interaction in order to infer the possible functions of the common DE-lncRNAs. However, the mechanisms by which the lncRNAs function should still be further explored by taking advantage of experimental approaches. Both microarray and RNA-seq are high-throughput technologies for reliable assessment of transcript abundance [51]. Microarray was chosen in this study for its low cost as well as flexibility compared with RNA-seq. Meanwhile, an integrative application of high-throughput sequencing data beyond level of gene expression, such as PARS-Seq [52] which enables genome-scale reconstruction of RNA secondary structure and CLIP-Seq [53] which allows detection of massive interacting RNAs for a specific protein, will contribute to better understanding of the functions and regulatory mechanisms of lncRNAs.

## CONCLUSIONS

In conclusion, we identified lncRNAs whose dys-regulated expression was shared among four types of cancer as well as lncRNAs whose expression was specifically active in specific type of cancer, suggesting potential contribution of lncRNAs to tumorigenesis and histogenesis of different tissues. In addition, due to the lack of sufficient knowledge about functions and mechanisms of the majority of lncRNAs, we inferred the possible functions of common DE-lncRNAs by establishing their association with PCGs, thus providing clues for further mechanism exploration by experimental approaches.

## MATERIALS AND METHODS

### Tissue samples

Paired cancer and adjacent non-cancerous tissues from 20 patients with gastric cancer, 20 patients with colon cancer, 16 patients with liver cancer and 20 patients with lung cancer were collected with informed consent from Chinese PLA General Hospital and Peking University Cancer Hospital & Institute. 30 paired samples of patients with liver, lung and colon cancer for external validation were gathered from First People's Hospital of Foshan. All samples were collected by surgical operation and quickly stored in −80°C.

### RNA extraction and reverse transcription

Frozen tissue was cut into 2−4mm^3^ for homogenization. Total RNA was isolated with TRIzol reagent (Invitrogen, 15596–018) according to the manufacturer's instruction. Genomic DNA was removed using recombinant DNase I system (Ambion AM2235, Ambion), The RNA quantity was measured by NanoDrop spectrophotometer (Thermo Scientific, USA) and the integrity was assessed using agarose gel electrophoresis, the 28S/18S ratio was about equal to 2.0. All steps were performed under RNase-free conditions.

5 ug total RNA of each sample was reverse transcribed into cDNA with the SuperScript III First-strand synthesis system (Invitrogen, 18080–051) using random hexamers following the manufacture's protocol.

### The custom designed microarray platform

The custom designed microarray platform was manufactured by Agilent, consisting of probes for 21,789 PCGs and 39,311 lncRNA transcripts. LncRNA transcripts were collected from a number of different sources including NONCODE [31], H-InvDB [54], UCSC, Ensembl [55], LincRNA Catalog [56] and so on (see Supplementary File 4 for details about lncRNA collection). At least one probe was designed for each lncRNA transcript. Of all of the probes designed for lncRNA transcripts, 28,937 are specific for lncRNA transcripts and do not overlap with protein coding loci. These lncRNA transcripts with unique probes were subjected to further analysis.

### Bioinformatics analysis of microarray data

The expression of lncRNAs and PCGs in obtained samples was examined using the microarray platform described above. Feature Extraction (Agilent Technologies, CA) software was used to extract all features of the data obtained from the scanned images. The lncRNA + mRNA array data were subjected for background subtraction and quality control by the GeneSpring software (Agilent). Quantile normalization was carried out on the whole set of probes for PCGs and unique probes for lncRNA transcripts for each type of tissues. Expression values were log2-scale transformed and then probes for mRNAs were collapsed down to gene level.

Hierarchical clustering was performed using cluster 3.0 [57] with complete linkage and centred Pearson correlation. The normalized and log2-scaled expression values were centred on the median before performing unsupervised hierarchical clustering. PCGs and lncRNAs were determined to be differentially expressed with two-tailed Student's *t*-test *p*-value < 0.05 (after FDR correction) and fold change greater than 1.5 between tumor samples and adjacent control samples.

### Construction of tri-color network

The construction of tri-color network consists of three steps: (i) prediction of miRNA targets of mRNAs and lncRNAs by miRanda; (ii) for each of the common DE-lncRNAs, calculate its expression profile correlation with its genomic nearest neighboring PCG; (iii) visualization of network by Cytoscape [58]. In the network, different types of RNAs were discriminated from each other by different colors (red, yellow, blue represents lncRNA, mRNA and miRNA respectively). Different color of edges represents different types of interactions. miRNA targeting of lncRNAs or mRNAs were represented by red lines while the genomic interaction and expression correlation between lncRNAs and mRNAs were represented by blue and light green respectively.

### Quantitative real-time PCR

Microarray data were validated by quantitative real-time PCR (qRT-PCR). The primers for validating selected genes were designed by software primer premier 5.0 and the IDT web server (http://sg.idtdna.com/Primerquest/Home/Index). The specificity of all the primers was confirmed by UCSC BLAT tool (http://genome.ucsc.edu/cgi-bin/hgBlat). All the primers were tested in Trans-Start top Green qPCR Supermix reaction (TransGen Biotech, AQ131–03) following the manufacture's protocol. The optimal primers were selected for quantitative validation.

The qRT-PCR assays were carried out on the Rotor-Gene Q real-time PCR cycler (Qiagen, 9001630) according to manufacturer's instruction. For each gene, qRT-PCR reactions were performed in technical triplicate, with 18S rRNA as internal control gene for normalization. The relative expression was calculated with the 2^−ΔΔCT^ method. The primer sequences were listed in Supplementary File 3.

### Functional enrichment analysis

Functional enrichment analysis of DE-PCGs were performed using the DAVID Bioinformatics Tool [59]. Gene set enrichment analysis was performed for common DE-lncRNAs as previously described [32]. For each lncRNA, the Pearson correlation coefficients with all PCGs based on their expression in all samples were calculated as the weight subjected to GSEA. Then the degree to which a specific pathway related genes were overrepresented was calculated as the enrichment score representing the correlation between the lncRNA and the pathways. A positive/negative enrichment score with higher value means higher positive/negative correlation between the lncRNA and the pathway. The GSEA software and the gene set database were downloaded from http://www.broadinstitute.org/gsea/.

### Accession numbers

The NCBI GEO accession number for the microarray data reported in this paper is GSE70880.

## SUPPLEMENTARY FIGURES AND TABLES




