# Supplementary material for: Transcriptional profiling analysis and functional prediction of long noncoding RNAs in cancer
Source: Oncotarget. 2016 Jan 23;7(7):8131–42. doi: 10.18632/oncotarget.6993 (PMC4884981; doi:10.18632/oncotarget.6993)
Supplement: Supplementary file 1 [file oncotarget-07-8131-s001.pdf]

Transcriptional profiling analysis and functional prediction of long noncoding RNAs in cancer

Supplementary Materials

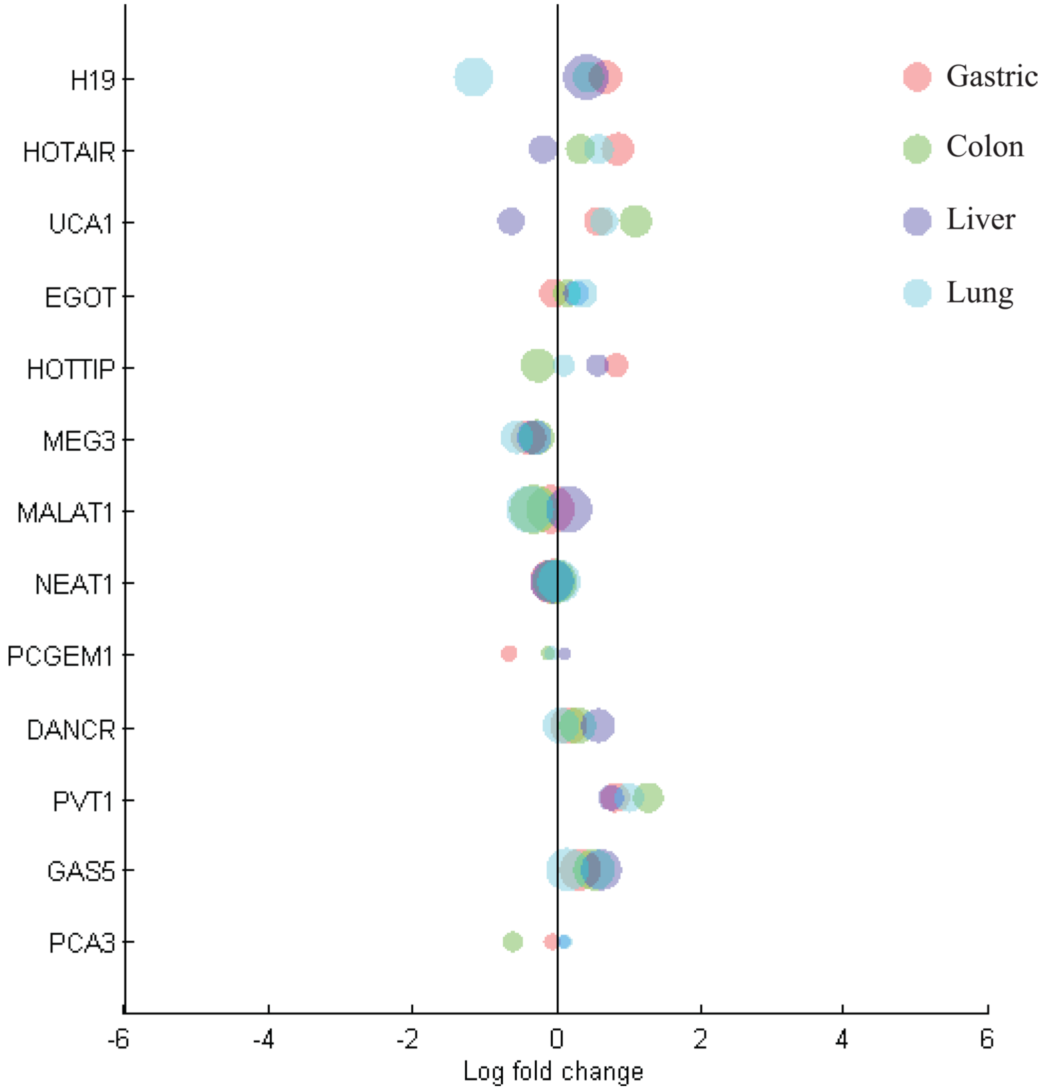

Supplementary Figure S1: Expression of literature-curated lncRNAs revealed by our data. Expression levels of literature-curated cancer-related lncRNAs across different types of cancer (tissues were denoted by different color). The size of each point represents the average expression level while the x-axis shows log2 fold change of expression level of cancer sample compared to adjacent non-cancerous sample.

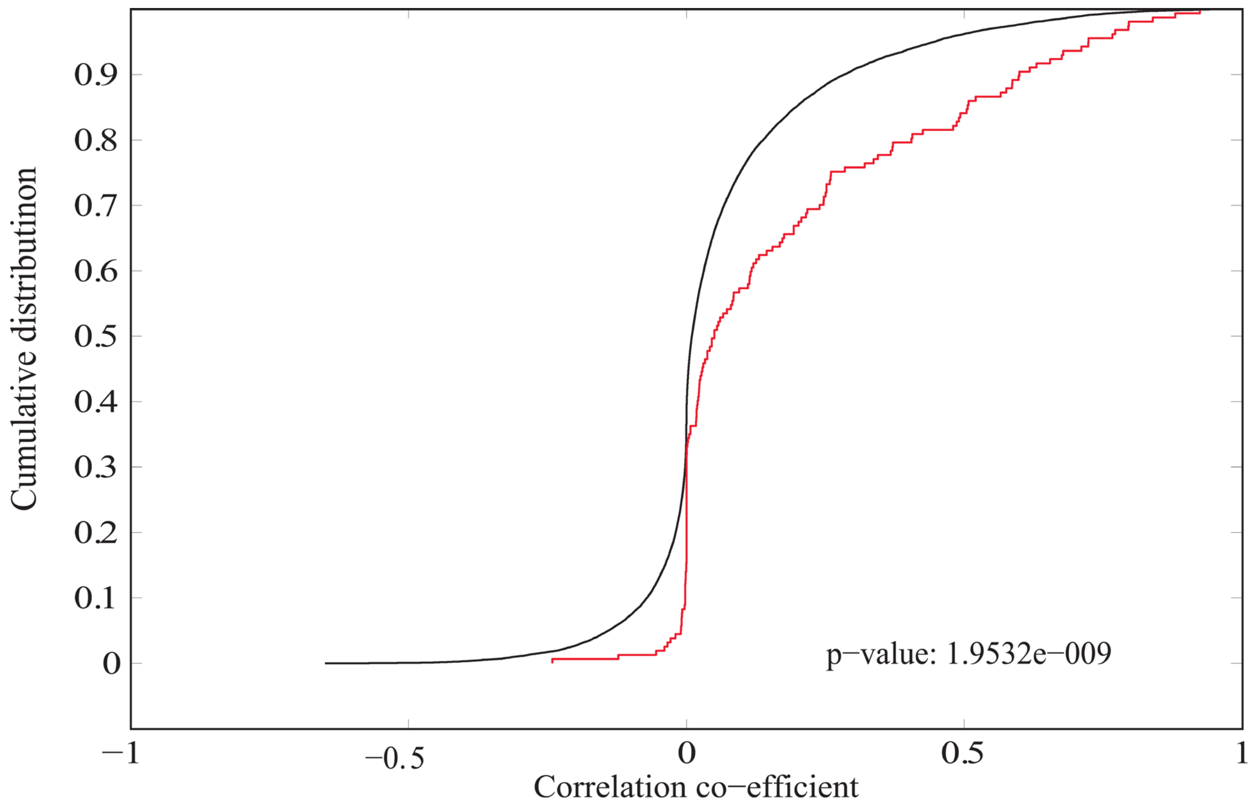

Supplementary Figure S2: Expression correlation analysis for lncRNAs and their neighboring PCGs. Empirical cumulative distribution plot of expression correlation coefficients for pairs of lncRNAs and their neighboring PCGs (red line for common DE-lncRNAs and black line for other lncRNAs as background). Rank-sum test showed significant difference between them, suggesting a whole tendency to be positive correlated for expression profile of common DE-lncRNAs and their neighboring PCGs.

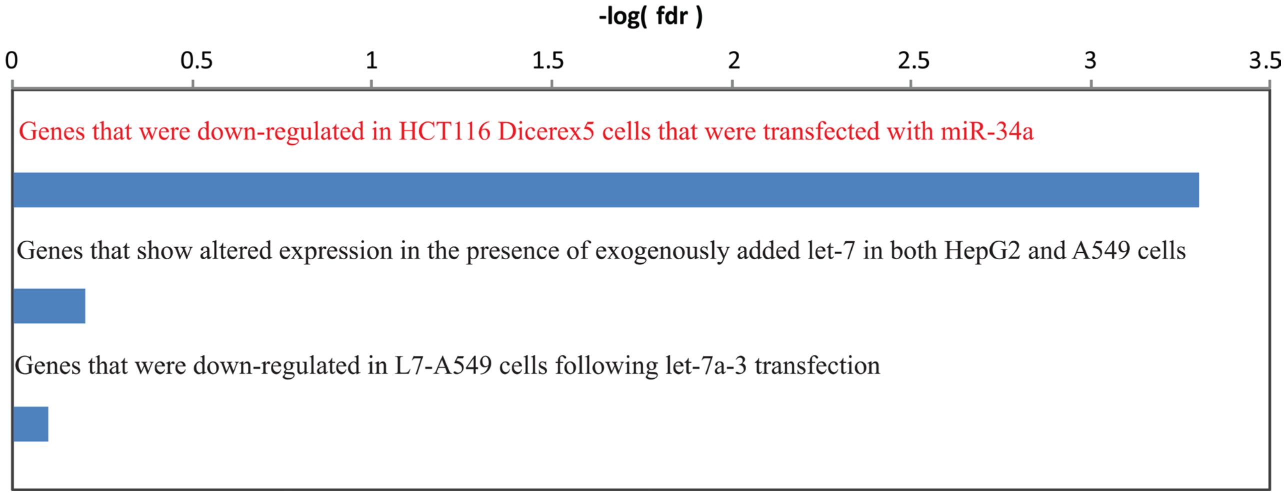

Supplementary Figure S3: miRNA target enrichment in cis mRNAs of lncRNAs. The height of each bar represents enrichment significance in the neighboring PCGs of common up-regulated lncRNAs calculated by GREAT (Genomic Regions Enrichment of Annotations Tool). The enrichment for miRNA targets ontology collected by Ron Shamir's group from the literature was presented.

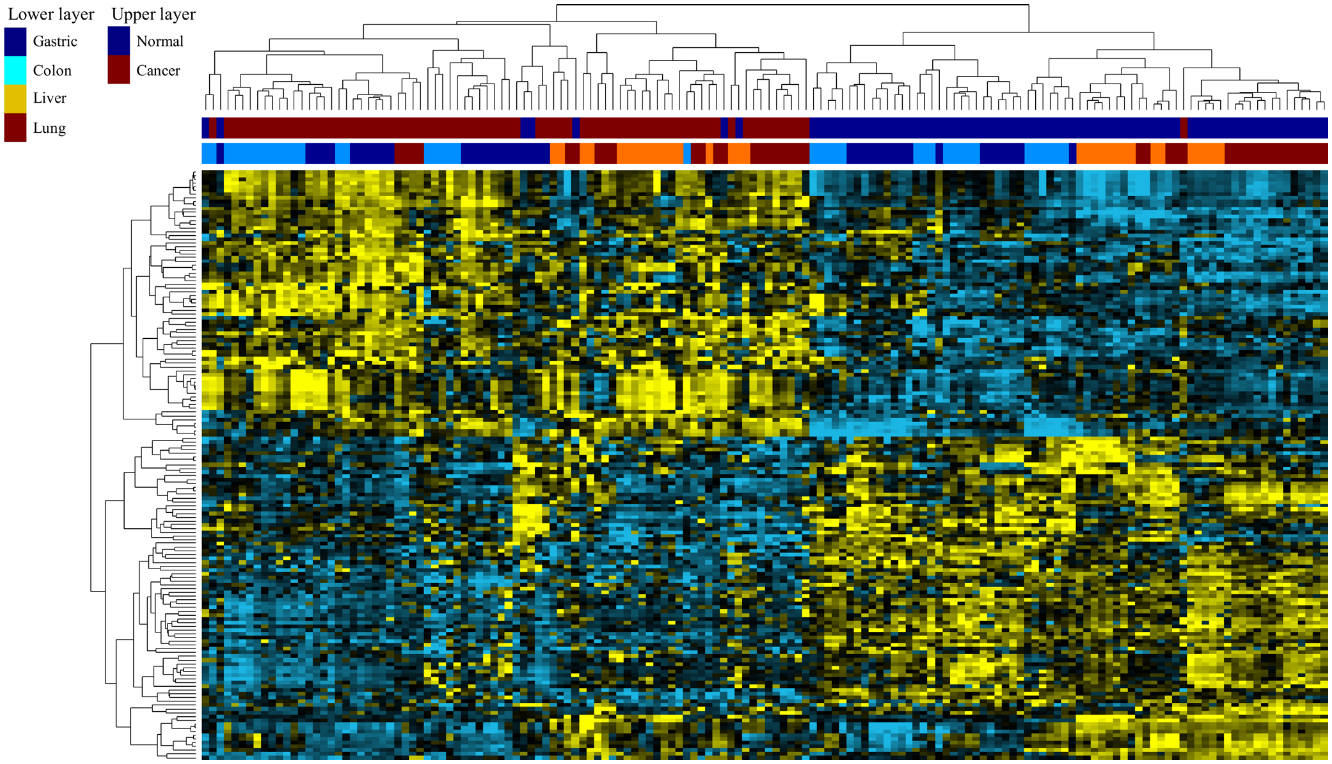

Supplementary Figure S4: Unsupervised hierarchical clustering of all samples. The expression profile of the 157 identified common DE-lncRNAs was used. Hierarchical clustering clearly separated tumor (red bar) and adjacent non-tumor (blue bar) samples with misclassification tolerated. Although different types of tissue were not clustered exactly, there is a tendency for liver tissues to be clustered with lung tissues, similar to that of gastric tissues with colon tissues.

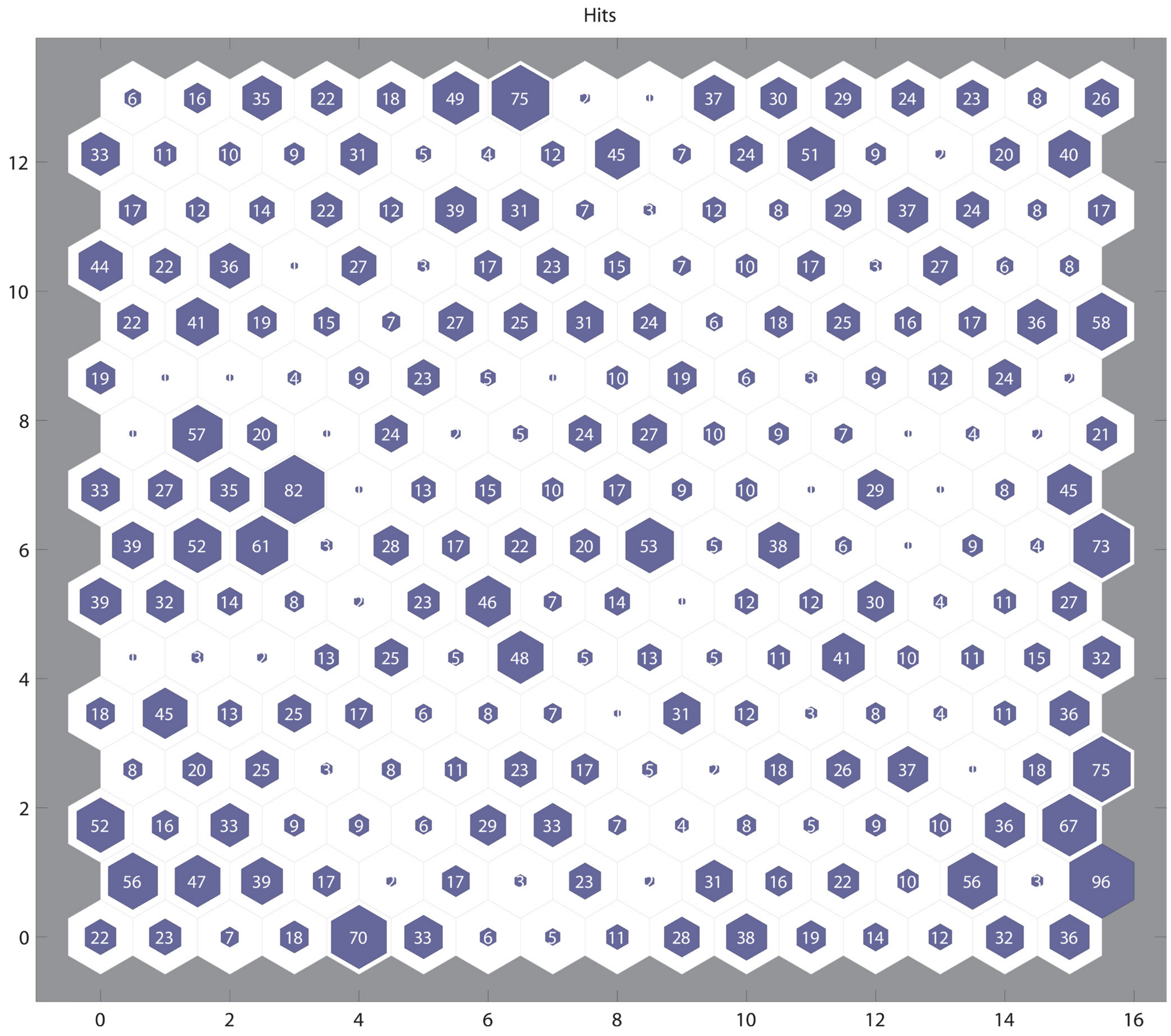

Supplementary Figure S5: The layout of SOM grids. Each grid is a cluster of lncRNAs and PCGs which are near each other not only in the SOM grid but also in multivariate space. The value shown in each grid represents the number of lncRNAs and PCGs clustered in the grid.

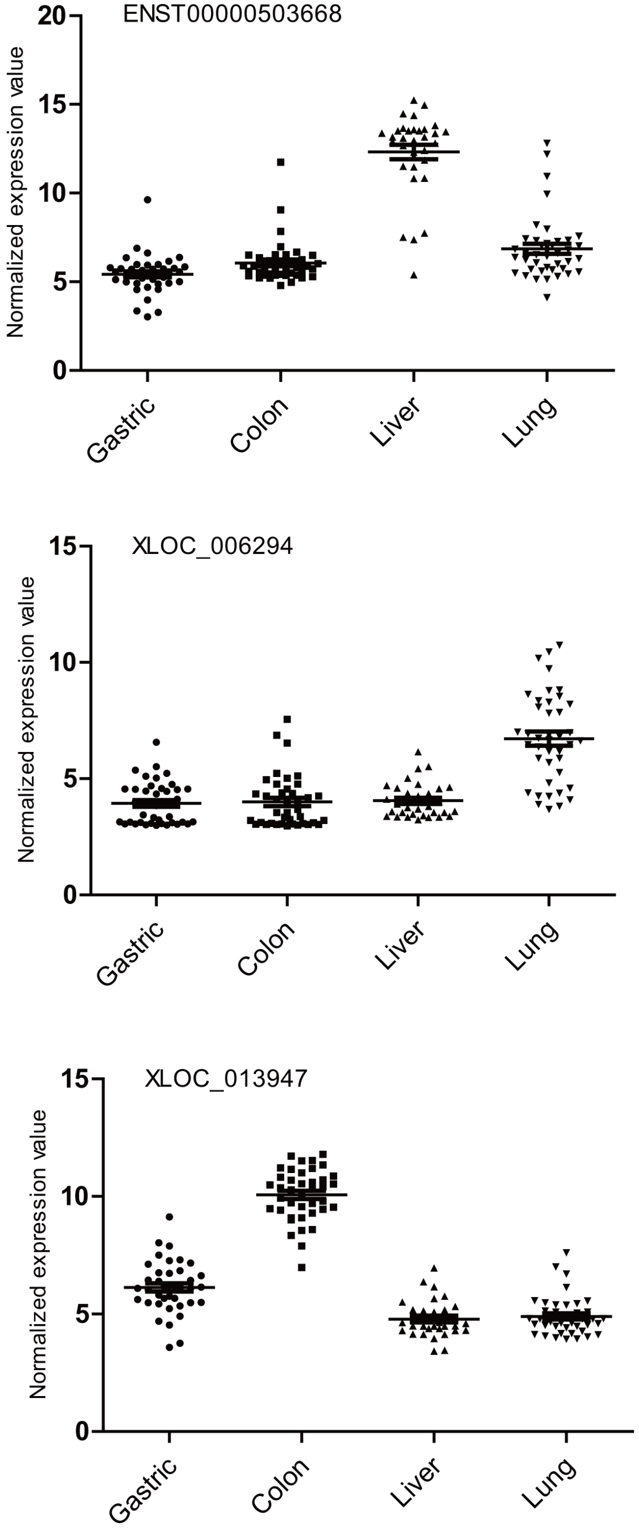

Supplementary Figure S6: Expression of tissue-specific lncRNAs based on the microarray data. Each scatter diagram illustrates the tissue-specific lncRNA possessing the higher expression level in specific tissue than the three others.

Supplementary File S1: List of DE-lncRNAs and DE-PCGs that are common in all four types of cancer

Supplementary File S2: List of DE-lncRNAs and DE-PCGs that show tissue specific expression features

GeneName	Gastric	Colon	Liver	Lung
A4GNT	0.450894	0.123839	0.174321	0.250946
AKR1B10	0.405057	0.26333	0.143103	0.18851
AKR1B15	0.410131	0.282336	0.154885	0.152649
ALDH3A1	0.422085	0.248022	0.102597	0.227296
ANXA10	0.44496	0.206914	0.215858	0.132267
ATP4A	0.437135	0.25817	0.103027	0.201668
ATP4B	0.442638	0.262642	0.139491	0.155229
C1orf129	0.437994	0.202098	0.130203	0.229704
C1orf49	0.434813	0.108617	0.149037	0.307534
C20orf114	0.432577	0.086085	0.151015	0.330323
C3orf57	0.428879	0.156605	0.180513	0.234004
C6orf58	0.45399	0.167871	0.150413	0.227726
CCKAR	0.416925	0.142501	0.134847	0.305728
CPA2	0.413227	0.230392	0.130633	0.225748
DAZ2	0.404713	0.139319	0.176213	0.279756
DAZL	0.398005	0.154627	0.173805	0.273564
DPCR1	0.454162	0.222394	0.114723	0.20872
FBP2	0.447626	0.245184	0.134675	0.172515
FMO6P	0.441348	0.150843	0.125129	0.28268
FUT9	0.427159	0.116701	0.147059	0.309082
GHRL	0.435329	0.165979	0.154025	0.244668
GIF	0.452098	0.244066	0.082559	0.221276
GKN1	0.455366	0.191348	0.136223	0.217062
KCNE2	0.443584	0.181975	0.148349	0.226092
LIPF	0.452356	0.144221	0.128397	0.275026
LOC100128893	0.407981	0.274338	0.160131	0.157551
LOC646804	0.437393	0.210956	0.139405	0.212246
LYPD6B	0.438338	0.262728	0.084279	0.214654
MIA	0.426127	0.265566	0.087633	0.220674
MUC6	0.449346	0.252322	0.144565	0.153767
NMUR2	0.427245	0.218008	0.071207	0.28354
PDIA2	0.446336	0.291366	0.091159	0.171139
PSAPL1	0.442724	0.101307	0.172687	0.283282
PSCA	0.455108	0.20227	0.089783	0.252838
REG3A	0.420967	0.302804	0.140695	0.135535
REG3G	0.420193	0.237014	0.140609	0.202184
SLC5A5	0.445648	0.234864	0.120485	0.199002

SULT1C2	0.451324	0.223598	0.117475	0.207602
SULT1C2P1	0.446766	0.225662	0.117733	0.209838
TFF2	0.455366	0.236326	0.146285	0.162023
UPK1B	0.430255	0.132439	0.132955	0.304352

$$score = \frac{\text{Sum of expression value in samples of specific tissue}}{\text{Sum of expression value in all samples}}$$

Supplementary File S3: List of primers used in this study

Gene Name	Primer Orientation	Primer Sequence(5'–3')
HJURP	Forward	CCTTCACGTTCAGGGAAATAG
HJURP	Rverse	CCAGGAGATTTGAGGCAATAC
RNA39743|RefSeq_1746_1992	Forward	GCAACTGTCCAACAAGGTTTAG
	Rverse	GGAACCGTCAGAAGCAAATTATAG
RNA33853|lncRNAdb_60_500	Forward	GGAGCCTTTACAAGGGAATG
	Rverse	GGATCACAGTTCTGGAGGT
RNA164968|XLOC_006294	Forward	GCAATGATGTTCCACAAGTTAG
	Rverse	CTGCAACCAGCATCTATGT
RNA162407|XLOC_013947	Forward	GCCAATGCAAGACCCATTTC
	Rverse	CCAGCAGATCTCTGTGTATGTC
18s rRNA	Forward	GAG GAT GAG GTG GAA CGT GT
	Rverse	TCT TCA GTC GCT CCA GGT CT

Supplementary File S4: The composition of transcripts collected in the custom-designed microarray

Source	Number
ENSEMBL	12,754
LincRNA Catalog	8,195
lncRNAdb	78
LNCipedia	–
RefSeq	4,765
UCSC	13,521
H-InvDB	17,203
NRED	1,289
lncRNAs from HOX loci	407
lncRNAs from UCRs	481
lncRNAs from literature	529
lncRNAs from IBP	848
lncRNAs with Enhancer-like Function	2,975
Antisense ncRNA pipeline	1,053
Non-coding RNA Search	1,273
snoRNA	389
Total	65,760
Total (non-redundant)	39,213
